# Multiple endocrine neoplasia type 1 presenting with concurrent insulinoma and prolactinoma in early-adolescence

**DOI:** 10.1186/s13633-018-0061-6

**Published:** 2018-08-06

**Authors:** Yasmin Akhtar, Angela Verardo, Janet L. Crane

**Affiliations:** Department of Pediatrics, Johns Hopkins University School of Medicine, 200 N Wolfe St, Rm 3120, Baltimore, MD 21287 USA

**Keywords:** MEN1, Prolactinoma, Insulinoma

## Abstract

**Background:**

Multiple Endocrine Neoplasia Type 1 (MEN1) is a rare autosomal dominant disease that generally presents with primary hyperparathyroidism. However, initial presentation may vary and continued reevaluation of etiology of symptoms is required for appropriate diagnosis.

**Case Presentation:**

Twelve year old female presented with altered mental status that self-resolved and hypoglycemia. Laboratory evaluation revealed pituitary dysfunction with central hypothyroidism and adrenal insufficiency in the setting of hyperprolactinemia. Macroadenoma was confirmed on imaging. Despite medical treatment of pituitary hormone disorders, she continued to have significant hypoglycemia and further workup revealed hyperinsulinism. Insulinoma was identified and confirmed by endoscopic ultrasound. Hypoglycemia resolved after laproscopic enucleation of the insulinoma.

**Conclusion:**

Children presenting with one endocrine tumor should be investigated for other potential endocrine tumors. Multiple imaging modalities may be required to confidently identify neuroendocrine tumors for appropriate surgical intervention.

## Background

In pediatrics, hypoglycemia most commonly presents in infants and toddlers. Presentation in early adolescence is unusual, but can be due to ingestions including hypoglycemic agents or ethanol or hormonal causes, such as adrenal insufficiency or hyperinsulinism. Congenital hyperinsulinism manifests early in life, whereas insulinomas develop later. Insulinomas are exceedingly rare, with an incidence of four cases per million per year, and are generally seen in an older population, with average diagnosis in the fifth decade of life [1]. Less than 100 pediatric insulinoma cases have been reported since the 1960s [2]. Insulinomas can be associated with Multiple Endocrine Neoplasia Type 1 (MEN1), though only 4–6% of patients with insulinomas have MEN1 [1].

MEN1 is diagnosed in an individual who has either two MEN1-associated tumors or one MEN1-related tumor with a first-degree relative with MEN1 or known MEN1 mutation. The most commonly impacted gland is the parathyroid, followed by the pancreatic islets and anterior pituitary [3]. Typical age of onset of MEN1 parathyroid adenoma is 25 years, gastrinoma 35 years, and prolactinoma 35 years [4]. The majority of MEN1 cases present initially with primary hyperparathyroidism (67%), whereas pituitary tumors and gastoenteropancreatic neuroendocrine tumors are less seldom the initial presentation [5]. Specifically, prolactinoma is the first identified MEN1 tumor in about 9.8% of cases, insulinomas 4.1% [5]. The case we describe is unique given simultaneous presentation with prolactinoma and insulinoma in an early-adolescent, leading to the diagnosis of MEN1.

## Case presentation

A previously healthy, premenarchal 12 year-old female presented to a local emergency department with altered mental status in the morning. She was back to baseline upon arrival except for recall deficit. Her initial point-of-care plasma glucose was 44 mg/dL. Serum glucose was confirmed low at 49 mg/dL. Family denied history of any hypoglycemic symptoms, except for occasional sluggishness in the morning. She had no signs/symptoms of infection and there were no medications in the home that could cause hypoglycemia. Family history was also negative for hypoglycemia or seizures. We recommended 24-h observation with frequent plasma glucose monitoring and additional laboratory evaluation. She continued to have hypoglycemia by point-of-care testing, requiring dextrose containing IV fluids overnight. Despite the fluids, AM plasma glucose was 58 mg/dL. Her 8 am cortisol was 2.2 mcg/dL, ACTH 30 pg/mL. TSH was normal at 2.169 mIU/mL, no free T4 resulted. Infectious workup and toxicology screen were negative, including oral hypoglycemic agents. Given the persistent hypoglycemia after 24 h and non-reassuring morning cortisol, we recommended transfer for additional evaluation.

**Fig. 1:**
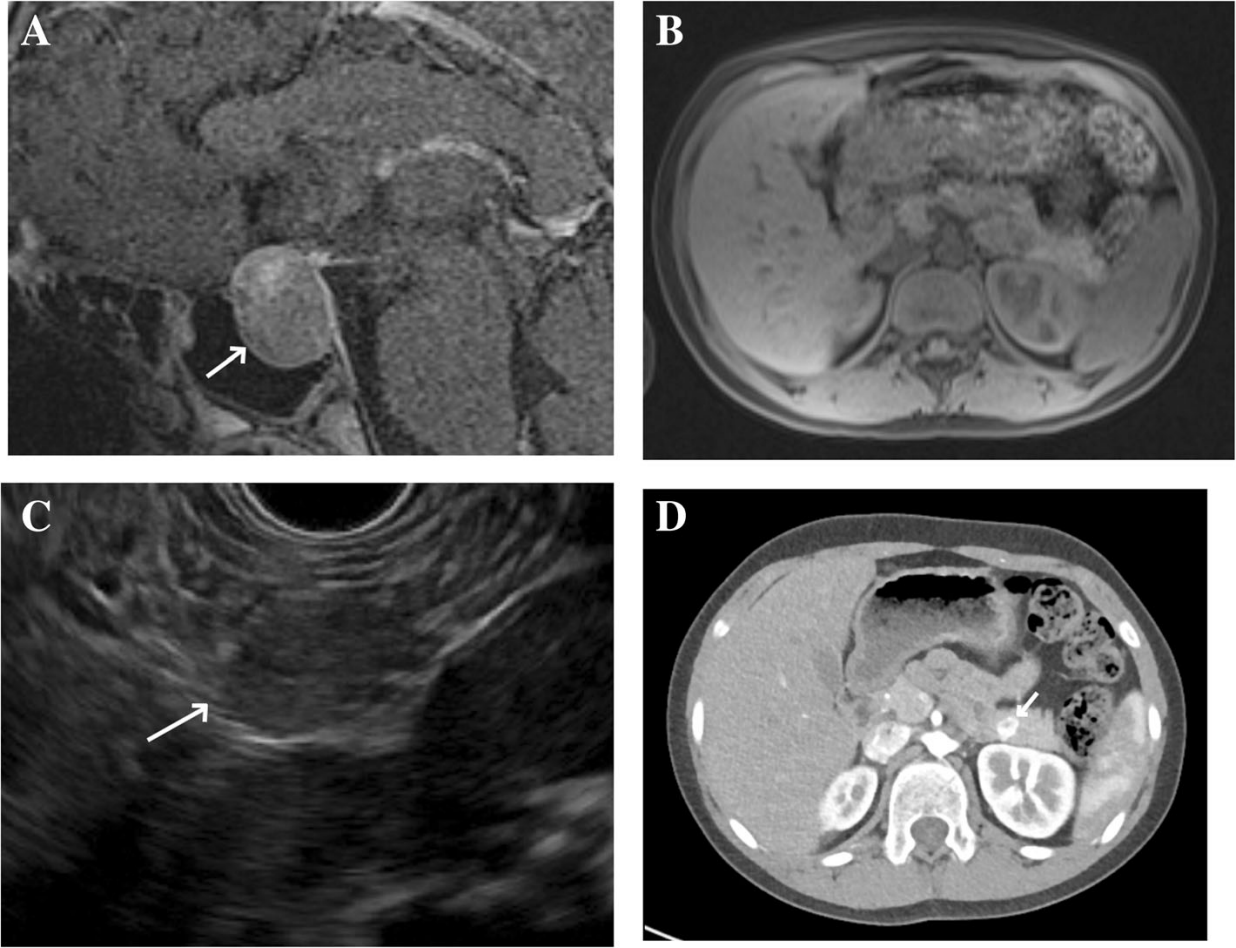
Imaging findings demonstrating pituitary macroadenoma and pancreatic adenoma. **a** MRI demonstrates enlarged pituitary extending into suprasellar cistern (white arrow) and elevating optic chiasm. **b** MRI of abdomen demonstrated normal pancreatic signal intensity without identification of any focal lesion or masses. **c** Endoscopic ultrasound revealed circumscribed hypoechoic mass (white arrow) in body/tail of pancreas. **d** CT image demonstrating hyperenhancing mass in distal pancreatic body (white arrow)

Her initial physical exam was normal (height 144.5 cm, 17th percentile for age; weight 39.5 kg, 38th percentile for age), visual fields intact, no signs of hyperpigmentation, Tanner II breasts (B2) with Tanner I pubic hair (PH1). Review of growth charts from her pediatrician did not demonstrate much change in height percentiles, growing around the 25th percentile for the last few years. ACTH stimulation test revealed cortisol of 1.3, 12, and 14.9 mcg/dL pre-, 30- and 60-min post-cosyntropin, respectively. Other pituitary hormones demonstrated a low free T4 of 0.5 ng/dL, normal FSH and LH and elevated prolactin level of 842.2 ng/mL (Table 1 for SI units). Brain and pituitary MRI showed a 2.0 × 1.5 × 1.9 cm enhancing mass expanding the sella turcica (Fig. 1a). She was initiated on hydrocortisone 6 mg/m^2^/day, increased to 12 mg/m^2^/day secondary to persistent hypoglycemia, levothyroxine 50 mcg daily, and cabergoline 0.25 mg twice weekly. Family was instructed on glucometer usage and advised to monitor blood sugars every morning and for concerning symptoms.

**Table 1:** Laboratory values

Lab	Conventional	SI
Insulin	13.6 μIU/mL at glucose 52 mg/dL	94.5 pmol/L @ glucose 2.89 mmol/L
	11.1 μIU/mL at glucose 48 mg/dL	79.6 pmol/L @ glucose 2.66 mmol/L
C peptide	2.7 ng/mL	0.9 nmol/L
Pro-insulin		18.1 pmol/L
beta-hydroxybutyrate	0.6 mg/dL	57.64 μmol/L
8 am Cortisol	2.1 μg/dL	58 nmol/L
8 am ACTH	30 pg/mL	6.6 pmol/L
TSH	2.169 mIU/mL	2.169 mIU/mL
Free T4	0.5 ng/dL	6.44 pmol/L
Cortisol s/p cosyntropin	1.3, 12, and 14.9 μg/dL pre-, 30- and 60-min post-cosyntropin	35.9, 331, 411.2 nmol/L pre-, 30- and 60-min post-cosyntropin
Prolactin	842.2 ng/mL	36.6 nmol/L

**Fig. 2:**
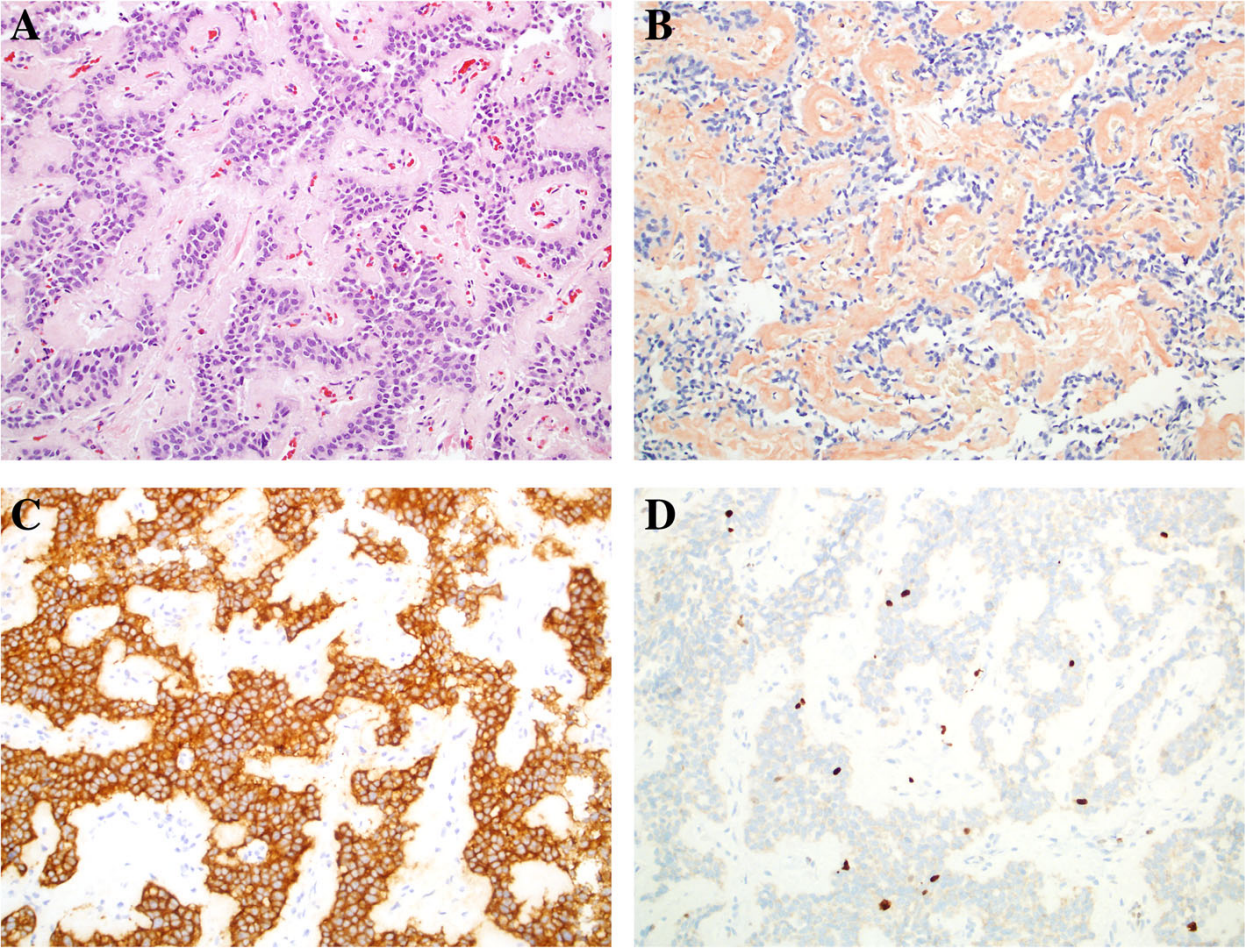
Immunohistochemical staining of pancreatic insulinoma. **a** Hematoxylin & eosin stain showing the tumor cells, **b** Congo red stain highlighting amyloid deposition, **c** Synaptophysin stain demonstrating neuroendocrine differentiation, and **d** Ki67 stain showing a proliferation index of 1–2%. Images acquired at 200X magnification

After discharge, she continued to have fasting hypoglycemia. Review of additional critical labs from the referral institution demonstrated an insulin level of 13.6 uIU/mL when serum glucose was 52 mg/dL. Two days after discharge, a second set of fasting critical labs were repeated at a local laboratory and were remarkable for an inappropriately elevated insulin concentration in the setting of hypoglycemia. When serum glucose was 48 mg/dL, insulin level was 11.1 uIU/mL, c-peptide was 2.7 ng/mL (normal range 1.1–4.4), proinsulin was 18.1 pmol/L (normal range 0–10), beta-hydroxybutyrate was 0.6 mg/dL (normal range 0.2–2.8), and urine was negative for ketones. Other pertinent labs included normal calcium levels, ranging from 9.2–9.3 mg/dL. Additional clinical interventions were recommended, including continuous glucose monitor, small frequent meals, cornstarch, and glucagon for emergency use. Imaging studies were pursued to localize the suspected insulinoma. Magnetic resonance imaging (MRI) of abdomen and pelvis with contrast was negative for any lesion or lymph node enlargement (Fig. 1b). Endoscopic ultrasound (EUS) identified a circumscribed 12 × 11 mm hypoechoic mass in the pancreatic body/tail (Fig. 1c). Immunostaining of a fine needle aspirate of the suspicious lesion was positive for chromogranin, synaptophysin, and insulinoma-associated-1 gene (INSM1) with 2–3% Ki-67 proliferation, confirming low-grade insulinoma (Fig. 2). Computed tomography (CT) of the abdomen was pursued for additional surgical planning, confirming presence of a 1.2 × 0.9 cm hyperenhancing mass in the distal pancreatic body with no evidence of metastatic disease (Fig. 1d). She underwent successful laparoscopic enucleation of the pancreatic lesion and has had no further hypoglycemia. Prolonged fasting revealed normoglycemia in the setting of ketonuria, confirming resolution of hyperinsulinemic hypoglycemia. She underwent genetic testing of *MEN1* to confirm the diagnosis. She was found to have a novel mutation, heterozygous for a single nucleotide duplication, c.1247dupT (Tyr417LeuX32). The duplication causes a frameshift with a premature stop codon at position 32 of the new reading frame. The truncation is expected to result in loss of nuclear localization signals and several modified residues and thus is considered to be pathogenic.

On follow-up, she recovered adrenal function as demonstrated by morning cortisol of 19.1 mcg/dL (527 nmol/L) 4 months after presentation. By 10 months after treatment of the prolactinoma, puberty had progressed slowly to Tanner B2PH2. Height initially declined to the 11th percentile at 4-month follow-up, but was back to the 19th percentile by 10 months, with a growth velocity of 11 cm/year. Repeat MRI 10 months after initiation of cabergoline demonstrated near-normalization of pituitary size (1.3 × 1.4 × 0.9 cm).

## Discussion

The average age at diagnosis of MEN1 in sporadic cases or a proband is 47.2 ± 15.3 years, whereas a family history results in diagnosis about 10 years earlier [5]. People without positive genetic testing are clinically diagnosed with MEN1 based on the presence of tumors in at least two MEN1 affected organs (most commonly parathyroid, pituitary, pancreas). As our patient presented with an insulinoma and prolactinoma, she met the clinical criteria of MEN1, which was confirmed by subsequent genetic testing. Diagnosis of an insulinoma in a pediatric patient is rare, but identifying two tumors at the time of diagnosis has not been previously reported in a child. On review of the literature, the majority of insulinomas diagnosed in childhood are identified secondary to a family history of MEN1. Only a few children presenting with an insulinoma have been the proband case with additional endocrine tumors identified years later through clinical monitoring [6–9]. Case reports of multiple endocrine tumors diagnosed simultaneously are often confounded by delayed diagnosis with many years of symptomatology [10, 11].

This patient initially presented with symptoms and evidence of hypoglycemia. Her work up revealed impairment of her hypothalamic-pituitary axes from mass effect of a prolactinoma. While there was no galactorrhea reported, she was on the late side of normal pubertal development. Post therapy, her pubertal status has progressed slowly. There was minimal suppression of her growth, noted after presentation with height declining slightly from the 17th to 11th percentile. Her height fell within her mid-parental height (around 25th percentile). Her continued hypoglycemia was most consistent with insulinoma that was confirmed biochemically and with imaging.

Following biochemical confirmation of hyperinsulinism with both an inappropriately elevated insulin level during hypoglycemia and an elevated proinsulin to insulin ratio, we were faced with the challenge of determining appropriate imaging. There is no consensus on first line imaging modality and our case confirms that this decision should be center specific. The least invasive methods include CT and MRI. As MRI and CT have comparable sensitivity (93 versus 73%, respectively) and specificity (88 versus 96%, respectively) [12], an MRI of the abdomen and pelvis was pursued first to avoid unnecessary radiation. The patient tolerated the procedure although reported difficulty in coordinating respirations with imaging acquisition. EUS was chosen as the next imaging modality, as it has a similar sensitivity and specificity relative to MRI and CT (93 and 95%, respectively) [12]. EUS also conferred the advantage of exploring possible extrapancreatic locations, increased ability to detect small lesions, and ability to biopsy suspicious lesions. Other modalities were considered such as somatostatin receptor scintiography and PET scan but were not utilized as the second line due to variable sensitivity (81–94%) and limited radioisotope availability [13]. CT, which also readily identified the insulinoma, was subsequently pursued to help optimize the surgical plan. Potential limitations of MRI have previously been reported where a patient with multiple pancreatic adenomas had only one visualized by MRI [14]. Our case continues to highlight that if there is a high clinical suspicion with biochemical confirmation of hyperinsulinism, case-by-case evaluation of which imaging modality to pursue is necessary. Furthermore, multiple imaging modalities may be necessary to confidently identify the insulinoma.

In conclusion, we report an early adolescent female with MEN1 after presenting with two endocrine tumors. Although the diagnosis of MEN1 is rare in this age group, particularly without a positive family history, a thorough evaluation is required for the appropriate diagnosis and treatment.

## Conclusions

Given the rarity of neuroendocrine tumors presenting in childhood, children presenting with one endocrine tumor should be evaluated for other potential endocrine tumors and consideration for genetic testing for neuroendocrine tumor syndromes. Multiple imaging modalities may be required to confidently identify neuroendocrine tumors for appropriate surgical intervention.
